# Breastfeeding, pregnant, and non-breastfeeding nor pregnant women's food consumption: A matched within-household analysis in India

**DOI:** 10.1016/j.srhc.2015.11.007

**Published:** 2016-03

**Authors:** Jasmine Fledderjohann, Sukumar Vellakkal, David Stuckler

**Affiliations:** a Department of Sociology, University of Oxford, Oxford, UK; b Public Health Foundation of India, Delhi, India

**Keywords:** NBP, Non-breastfeeding, non-pregnant women, NRHM, National Rural Health Mission, NFHS, National Family Health Survey, Breastfeeding, Pregnancy, India, Women's health, Nutrition

## Abstract

**Objective:**

Promoting breastfeeding is major maternal and child health goal in India. It is unclear whether mothers receive additional food needed to support healthy breastfeeding.

**Methods:**

Using the latest National Family and Health Survey (2005–2006), we applied multilevel linear regression models to document correlates of nutrition for (n = 20,764) breastfeeding women. We then compared consumption of pulses, eggs, meat, fish, dairy, fruit, and vegetables across a sample of breastfeeding, non-breastfeeding/pregnant (NBP), and pregnant women (n = 3,409) matched within households and five-year age bands. We tested whether breastfeeding women had greater advantages in the 18 high-focus states of India's National Rural Health Mission (NRHM).

**Results:**

Vegetarianism, caste, and religion were the strongest predictors of breastfeeding women's nutrition. Breastfeeding women had no nutritional advantage compared to NBP women, and were disadvantaged in their consumption of milk (b = −0.14) in low-focus states. Pregnant women were similarly disadvantaged in their consumption of milk in low-focus states (b = −0.32), but consumed vegetables more frequently (b = 0.12) than NBP women in high-focus states.

**Conclusions:**

Breastfeeding women do not receive nutritional advantages compared to NBP women. Targeted effort is needed to assess and improve nutritional adequacy for breastfeeding Indian women.

## Highlights

• Mothers require additional calories to support healthy breastfeeding. It is unclear whether this additional intake need is being met, especially in India where undernutrition is high
• We matched women within households to compare food consumption frequency
• Breastfeeding women are not nutritionally advantaged in the household across food items, and are disadvantaged in their access to milk
• Breastfeeding women's nutrition should receive greater programmatic attention in India

## Introduction

India is in the midst of a rapid nutrition transition, characterized by high rates of malnutrition and rising obesity [1]. There is a massive push in global health to promote breastfeeding to help tackle these concerns, as well as to improve cognitive development and reduce infectious disease risk [2], [3], [4], [5]. Although the health benefits of breastfeeding are debated in high-income countries [6], [7], there is virtually a consensus that breastfeeding is positive for children's development in low- and middle-income settings [8], [9]. Save the Children argues that “Mother's milk is effectively a child's first vaccination – and can often be the difference between life and death…In fact, mother's milk is the best food for the baby” [3], and both WHO and UNICEF recommend exclusive breastfeeding for the first 6 months of life, and complementary feeding for at least two years thereafter [4].

The effectiveness of breastfeeding, however, depends significantly on the state of mothers' nutrition. As nutritional needs increase during pregnancy and lactation [10], [11], [12], an increase in food consumption is necessary. Macro- and micro-nutrient deficiencies in breastfeeding women may lead to a reduction in the micronutrient and caloric content of breast milk [11], [13]. This is especially important in India, where it is estimated that around half of women are anaemic [14] and one-third are underweight [15], representing one of the highest rates of maternal malnutrition in the world. Mothers' malnourishment has also been linked to children's immunological development and survival, even if they are able to breastfeed [16], [17]. In spite of the tremendous importance of maternal nutrition during breastfeeding, the predictors of breastfeeding women's nutrition have not been documented. Recent work suggests that there is a socioeconomic gradient in propensity to breastfeed in a Western setting, but that the gradient is not observable among migrants from middle-income countries [18]. Whether there is a socioeconomic gradient in maternal nutrition is unclear, particularly in middle-income settings. Moreover, there is a dearth of literature investigating intra-household disparities in allocation of food and nutritional resources to breastfeeding women. In India, women's nutritional intake is of particular importance in light of rising food prices following the global recession [19], high rates of maternal malnutrition [20], and evidence of food allocation biases within the household [21], [22].

The National Rural Health Mission, India's flagship government program to improve maternal and child health, was introduced in 2005. The program did not explicitly subsidize maternal nutrition, but may have improved nutrition and enhanced opportunities for effective breastfeeding by (1) providing subsidies for healthcare, which may increase the overall financial resources of the household, and, thereby, quantity and quality of available food; (2) improving mothers' access to healthcare, where nutritional counselling associated with regular check-ups may increase awareness of maternal dietary needs during both pregnancy and lactation; and (3) establishing monthly Village Health and Nutrition Days, wherein women can obtain nutritional counselling (amongst other services).

A handful of studies have examined the importance of dietary intake during pregnancy for ensuring maternal, foetal, and infant health [23], [24], [25], but empirical evidence on dietary intake during breastfeeding has been scarce. Here, we draw on the large sample size of India's National Family Health Survey to (a) document the sociodemographic correlates of food consumption among breastfeeding women and (b) test whether breastfeeding women are more likely to receive higher quality (and more costly) foods than women who were neither breastfeeding nor pregnant (hereafter NBP), matched within households and by 5-year age bands. As a secondary objective and point of comparison, we also examine pregnant women's nutrition. In light of the push for breastfeeding in particular, we examine whether mothers are in fact receiving a much-needed nutritional advantage. Although increased consumption across a variety of food items is necessary to produce high-quality breast milk [10], [26], we hypothesize that breastfeeding women will receive additional low-cost items (such as eggs and vegetables) compared to NBP women, but will not receive additional high-cost items, such as meat and fruit – that is, due to affordability concerns, households may recognize the nutritional needs of breastfeeding women, but may attempt to meet these needs through additional low-cost calories (quantity of food) rather than high-cost nutrients (quality of food). Additionally, we hypothesize that net of household resources, breastfeeding women in areas targeted for intervention by India's National Rural Health Mission (NRHM) – that is, high-focus states – will be more likely to receive a dietary advantage than breastfeeding women in low-focus states as a result of interventions targeting healthcare access and nutrition education.

## Methods

We utilize secondary nationally representative data from Round 3 (collected December 2005 to July 2006) of the National Family and Health Survey (NFHS-3), India's Demographic and Health Survey (DHS). These data, the most recently available, were obtained from a third party [27], and were completely anonymized prior to download; no ethics board review was required. The NFHS includes information on a wide variety of household and individual level variables, including reproductive histories, breastfeeding, food item consumption for mothers and children, and anthropometry. Following standard DHS data collection design, data were collected using a multistage stratified design across 29 states and territories [28]. The full sample includes 124,385 married and unmarried women aged 15–49. In our first set of models, documenting predictors of breastfeeding women's nutrition, we restricted our sample to the (n = 20,764) currently breastfeeding women in the data. Models for meat and fish consumption (n = 15,385) excluded vegetarians.

In our comparative models, we employed a matched design to correct for potential endogeneity, focusing our analysis on a subsample of women of the same age in the same household. Unlike methods comparing consumption between households, this matched design allowed us to compare women facing the same household resource constraints. The subsample was restricted to households with at least one pregnant or breastfeeding woman and one NBP woman in the same 5 year age band; households with only one woman, without at least two women in the same age band, or with no pregnant or breastfeeding women, were dropped. In a small subset of cases, 2 NBP women were matched to 1 breastfeeding (92 households) or pregnant woman (90 households). Similarly, there were 22 households with 2 breastfeeding women matched to 1 NBP woman, and 5 households with 2 pregnant women matched to 1 NBP woman. Additionally, because there were no pregnant women over the age of 40 and only 5 breastfeeding women over age 40 in the subsample, comparisons for these groups were not possible; women aged 40 and over were dropped, resulting in a final matched sample of (n = 3,409).

The sample was further disaggregated by pregnancy and breastfeeding status, and vegetarians were excluded from models of meat and fish consumption. For models comparing breastfeeding (n = 1,314) and NBP women (n = 1,244), pregnant women were excluded, resulting in a sample of 2558 (n = 1,737 with vegetarians excluded); similarly, breastfeeding women were excluded from models comparing pregnant (n = 463) to NBP women (n = 503 in full models; total n = 966; n = 694 with vegetarians excluded). Missing data (<1% for all variables included in the analyses) were handled using listwise deletion; 7 additional cases were dropped due to missing values on one or more of the consumption measures (n = 3,402).

### Analytic strategy

The dependent variables used in the analysis are based on self-reported frequency of consumption of each of 7 food items separately (milk or curd; pulses or beans; green leafy vegetables; fruit; eggs; fish; chicken or meat). Specifically, women were asked “How often do you yourself consume the following food items: never, occasionally, weekly, or daily?” Higher values indicate more frequent consumption, with values ranging from 0 (never) to 3 (daily consumption). In order to control for unobserved heterogeneity within the household, we applied multilevel linear regression models to examine the association between breastfeeding status and food consumption frequency:

$$\mathrm{Nutritio}n_{i,j}=\alpha +\beta_{\text{Sociodemographicsi},j}+\beta_{\text{HouseholdCharacteristicsi},j}+\mu_{i,j}+\varepsilon_{i,j}$$

Here, *i* is the woman and *j* is the household. Women's self-reported consumption of food items is represented by Nutrition on the left-hand side. Sociodemographics refers to women's age (years), education (years), parity (continuous), vegetarianism (vegetarian = 1), caste^1^ (dummies for scheduled caste, scheduled tribe, and other backwards caste; reference group other/no caste), religion (dummies for Muslim, Christian, and other religion; reference group Hindu), age of the breastfeeding child in months (in the non-comparative models only), and marital status (married = 1). HouseholdCharacteristics includes an index of household wealth created by the NFHS and widely utilized in extant research, and place of residence (urban = 1). *µ* is the household effect, and *ε* is the error term. The models for the full sample also included a set of 28 state dummies to account for between-state differences that might impact consumption patterns; state dummies were not included in the matched sample due to data constraints.

First, we examined sociodemographic predictors of breastfeeding for the full sample of (n = 20,764) breastfeeding women. The comparative models estimate consumption for breastfeeding versus NBP and, separately, pregnant versus NBP women using the matched sample of (n = 3,402). In the comparative models, coefficients for each food category (milk, pulses, etc.) represent the coefficients for pregnancy/breastfeeding status drawn from separate regressions for each food item. Additional analyses split the sample into high- and low-focus states based on their designation in the NRHM, the aforementioned government programme aimed at improving access to and quality of maternal healthcare [30]. Supplementary Table S1 provides a list of high and low-focus NRHM states. All analyses were performed using Stata version 13.1.

## Results

### Breastfeeding women's food consumption

Supplementary Table S2 provides the results of the models predicting breastfeeding women's consumption across the 7 categories of food. Controlling for other sociodemographics, household characteristics, and state/territory, the strongest predictors were vegetarianism, religion, and caste. Vegetarianism was significantly, negatively associated with frequency of milk (b = −0.22; SE: 0.02), fruit (b = −0.08; SE: 0.02), and particularly egg (b = −2.09; SE: 0.02) consumption. Compared to Hindus, Muslims reported a higher frequency of milk (b = 0.10; SE: 0.03) and pulse (b = 0.10; SE: 0.01) consumption, but a lower frequency of fish (b = −0.05; SE: 0.02) and meat (b = −0.11; SE: 0.02). Christians more frequently consumed pulses (b = 0.07; SE: 0.03) and fish (b = 0.17; SE: 0.03), but reported less frequent consumption of milk (b = −0.17; SE: 0.04) and vegetables (b = −0.10; SE: 0.02) than Hindus. Other religious groups ate pulses more frequently (b = 0.11; SE: 0.03), but fish less frequently (b = −0.11; SE: 0.04). Compared to those reporting other or no caste, scheduled castes consumed milk (b = 0.11; SE: 0.02), pulses (b = 0.04; SE: 0.01) and eggs (b = 0.07; SE: 0.02) more frequently, while scheduled tribes consumed pulses (b = 0.11; SE: 0.02), vegetables (b = 0.03; SE: 0.02), eggs (b = 0.07; SE: 0.02) and fish (b = 0.08; SE: 0.02) more frequently, but milk (b = −0.18; SE: 0.03) and fruit (b = −0.05; SE: 0.02) less frequently. Other backward caste members reported more frequent consumption of pulses (b = 0.04; SE: 0.01) and fruit (b = 0.05; SE: 0.02).

### Matched sample descriptive statistics

Table 1 provides the descriptive statistics for the matched sample for breastfeeding, pregnant, and NBP women. Breastfeeding women tend to be older, are more likely to be married, and have lower parity on average than women who are not breastfeeding. Patterns across other sociodemographic indicators are quite similar across groups, e.g. respondents have around 6–7 years of education on average, 30% are vegetarians, 70% are Hindu. However, NBP women report consuming milk more frequently (mean score 1.71) than breastfeeding (1.64) or pregnant (1.66) women. Consumption appears to be similar across groups for pulses, vegetables, eggs, and meat, while pregnant women appear to consume fruit less frequently (2.11) than breastfeeding (2.28) or NBP (2.30) women. Conversely, pregnant women consume fish more frequently (1.62) than breastfeeding (1.49) or NBP (1.46) women.

### Comparative models for breastfeeding women

**Fig. 1:**
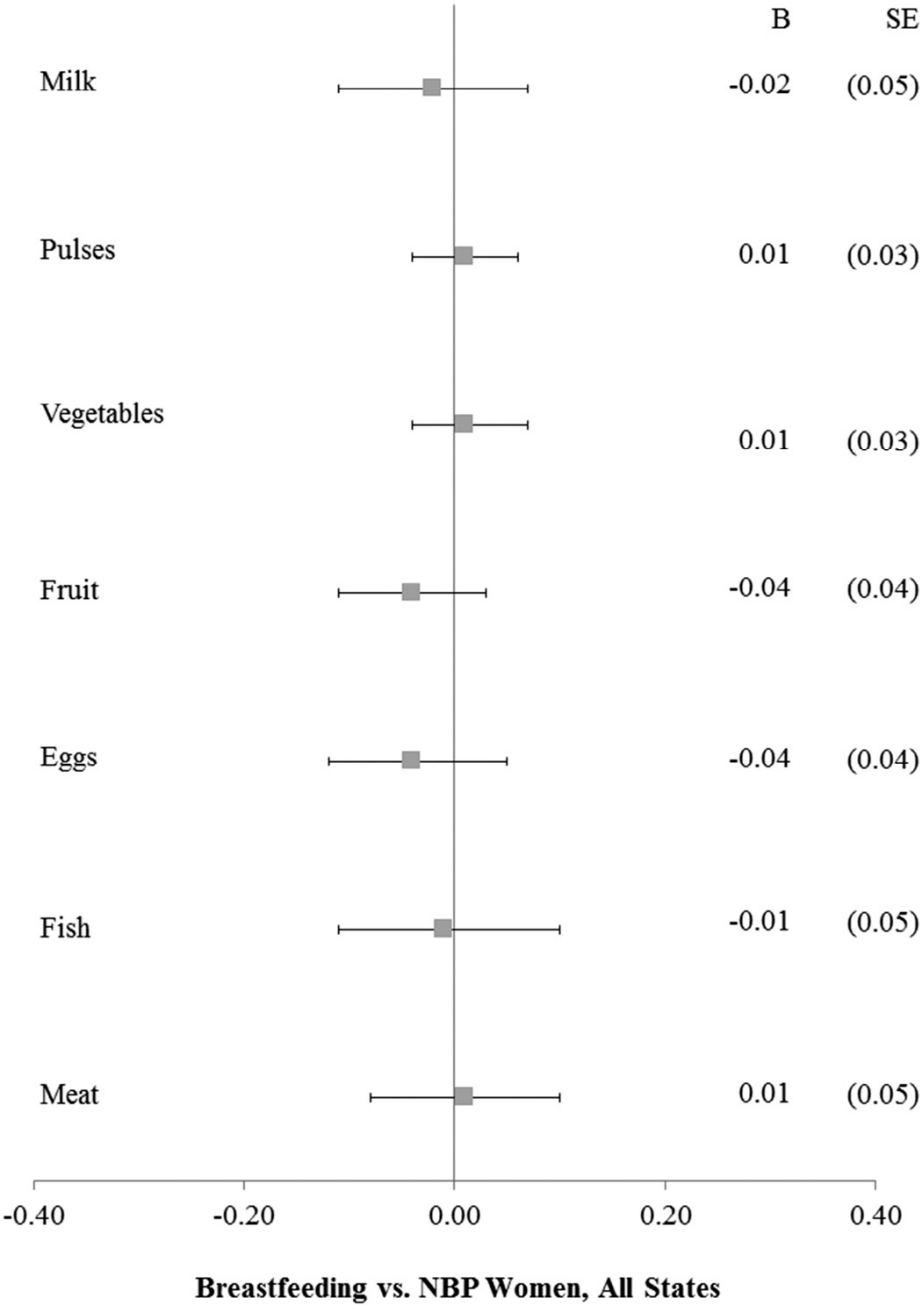
Food consumption for breastfeeding versus NBP women, matched sample, NFHS-3.

Applying multilevel linear models to the matched pair sample, we compared breastfeeding and NBP women's consumption patterns correcting for household and individual sociodemographic characteristics. Although coefficients for all covariates are not shown for brevity, a representative example of the full model is provided for fruit consumption in Supplementary Table S3. As shown in Fig. 1, no significant differences in frequency of consumption were detected across the 7 foods we examined. To test whether women in high-focus NRHM states, which greatly expanded maternal health programs, would receive greater food advantages than in low-focus states, we next split the sample into 18 high-focus and 11 low-focus states.

**Fig. 2:**
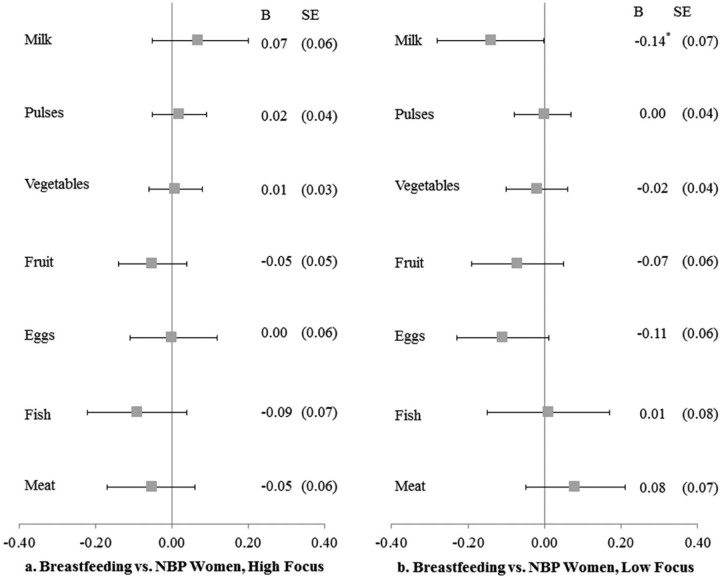
Food consumption for breastfeeding versus NBP women, high- and low-focus NRHM states, matched sample, NFHS-3.

Fig. 2 shows the forest plots for high and low-focus states separately. As shown in Fig. 2a, there were no significant differences for breastfeeding compared to NBP women in food consumption across categories in high-focus states. However, as Fig. 2b shows, breastfeeding women in low-focus states consumed milk less frequently (b = −0.014; SE: 0.07) than NBP women. No significant differences were observed for pulses, vegetables, fruit, eggs, fish, or meat.

### Comparative models for pregnant versus NBP women

**Fig. 3:**
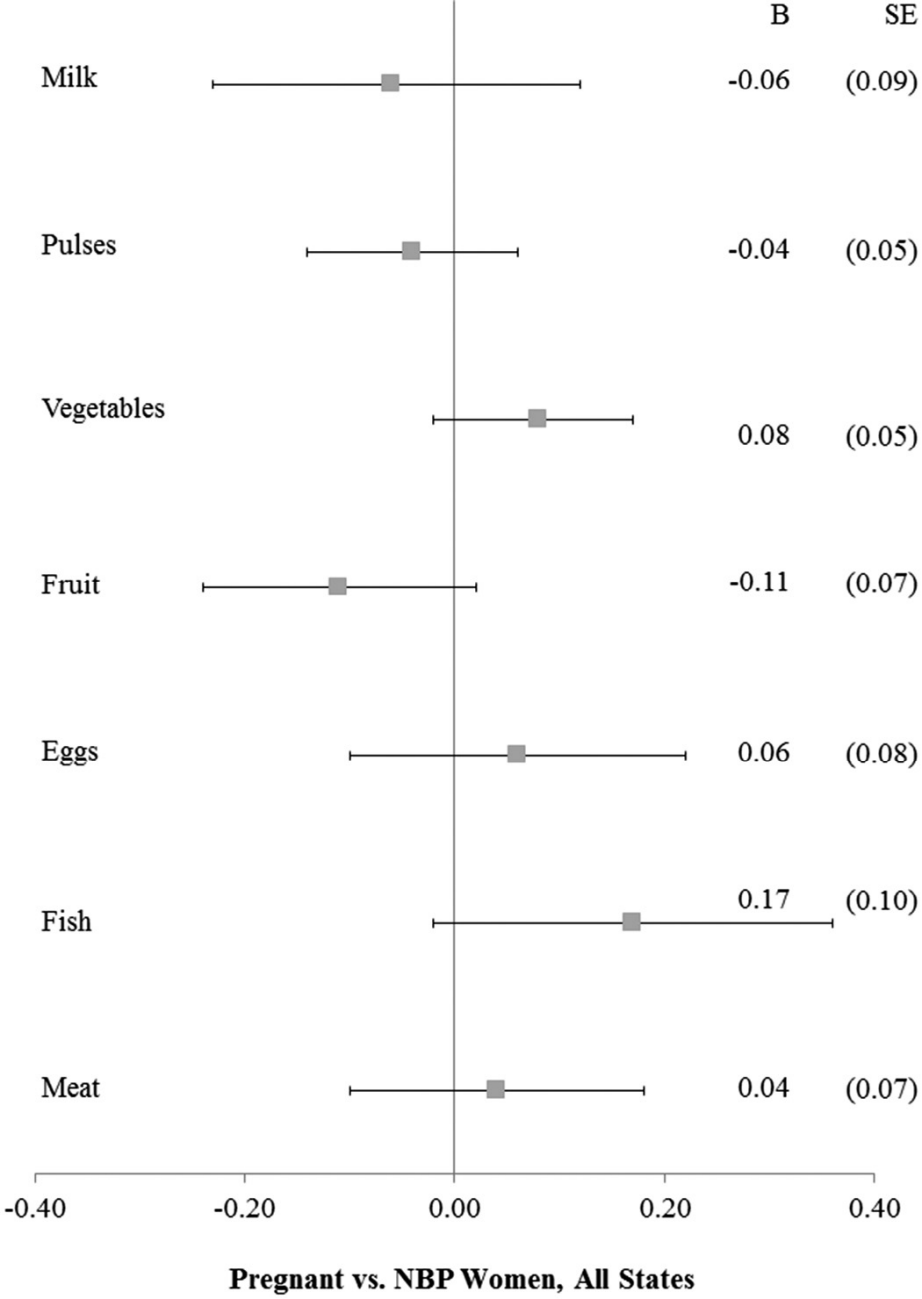
Food consumption for pregnant versus NBP women, matched sample, NFHS-3.

**Fig. 4:**
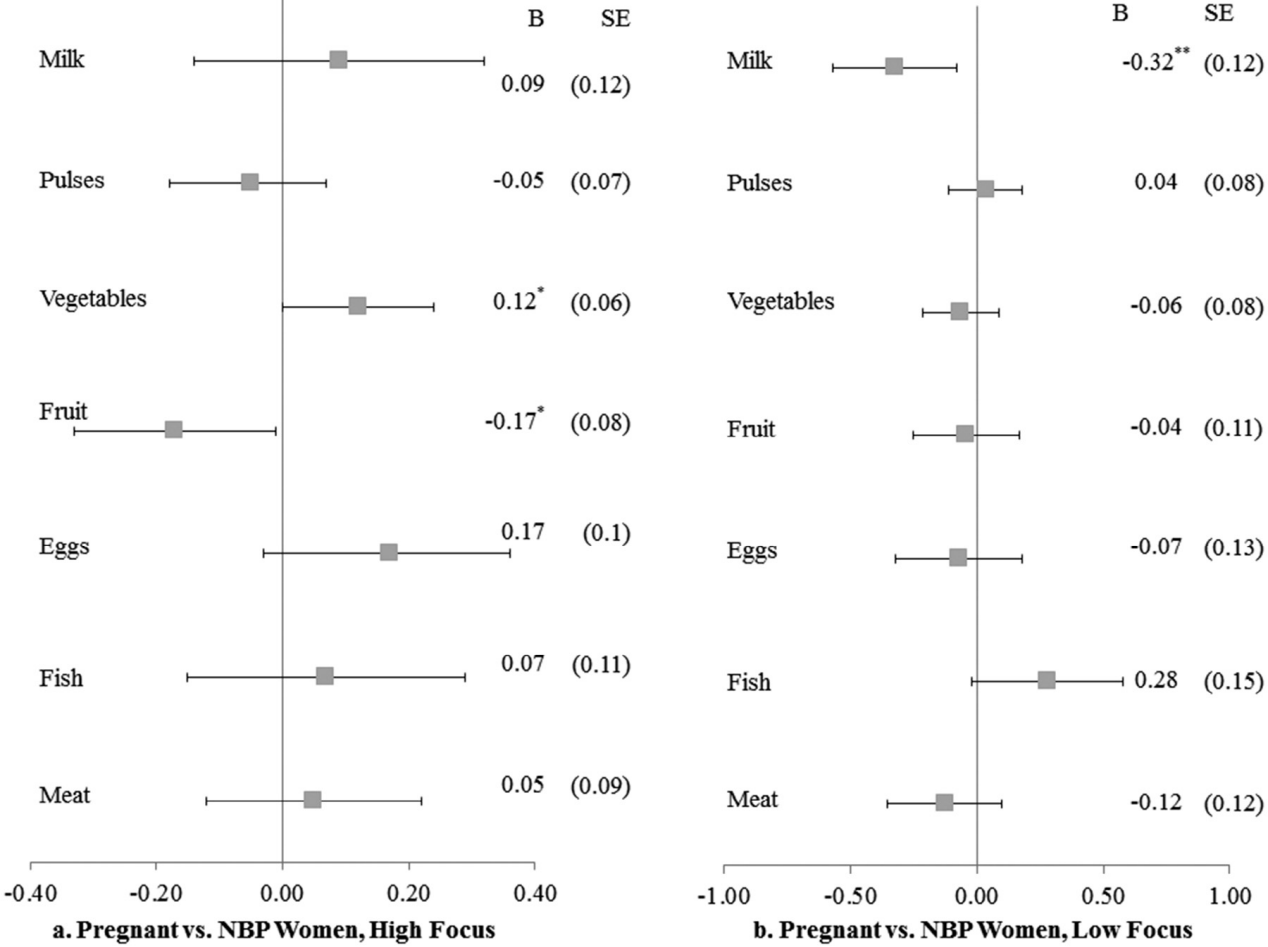
Food consumption for pregnant versus NBP women, high- and low-focus NRHM states, matched sample, NFHS-3.

In the final set of multilevel models, we compared pregnant women to NBP women to test the possibility that pregnant women were likely to receive additional and high-quality, higher cost food items within households. Fig. 3 shows the forest plots for pregnant women's food consumption compared to NBP women in all states. As with breastfeeding women, we found no significant differences between pregnant and NBP women's consumption. However, when we disaggregated by NRHM focus, shown in Fig. 4, we found that in high-focus states (Fig. 4a), pregnant women consumed vegetables more frequently (b = 0.12; SE: 0.06) but fruit less frequently (b = −0.17; SE: 0.08) than NBP women. In low-focus states, pregnant women reported lower milk consumption (b = −0.32; SE: 0.12) compared to NBP women.

### Robustness checks

Although we corrected for potential confounders in our models by controlling for household wealth and other sociodemographic characteristics, we further adjusted for State Domestic Product, taken from the Indian Government Directorate of Economics and Statistics [31], as a robustness check on our findings. The results of the models including State Domestic Product as an additional control variable are shown in Supplementary Table S4. None of the main results were qualitatively changed.

We employed multilevel linear regression models under the assumption that the latent construct we aim to measure, frequency of consumption, is in fact a continuous outcome. It is possible to using linear regression for ordinal outcomes under the continuous latent construct assumption and, so long as the linear specification does not alter substantive conclusions compared to the logit models, linear models are preferable [32], [33]. As a robustness check on our findings, and in order to validate the assumption that our linear models do not differ substantially from ordered logistic regression, we re-estimated all of our models using multilevel ordered logistic regression models. For brevity, we do not present the full set of results here.

While there were some marginal shifts in statistical significance owing to small cell sizes at the margins in some models, none of the results were substantially changed. For example, comparing breastfeeding women to NBP women in all states, the ordered logistic regression models show that there is a small, non-significant association, with breastfeeding women having slightly lower odds (OR = 0.95; 95% CI: 0.76 to 1.19; p = 0.67), while the linear model also shows a slight, non-significant, negative association (b = −0.02; SE: 0.05; p = 0.69). Moreover, as seen in this example, the confidence intervals are narrower in the linear than the ordered logistic models. As a second example, in low-focus states, compared to NBP women, breastfeeding women had a marginally non-significant coefficient indicating lower odds of receiving milk (OR = 0.72; 95% CI: 0.52 to 1.01; p = 0.05), while in the linear models this association is in the same direction and marginally significant (b = −0.14; SE: 0.07; p = 0.04).

## Discussion

In this study, we documented the predictors of breastfeeding women's food consumption using a nationally representative sample of Indian women, and used matched intra-household pairs of women to test whether breastfeeding women are given preferential access to household nutritional resources. This unique design allowed us to compare women facing similar household resource constraints in order to assess distributional inequalities within the household context. In the full sample, we found that vegetarianism, religion, and caste were the strongest predictors of women's food consumption. In the matched pair subsample, we found no evidence that breastfeeding women are advantaged compared to NBP women in the same household, and in fact found that, in low-focus states, they are disadvantaged in their consumption of milk. Pregnant women in low-focus states are similarly disadvantaged in their consumption of milk, and they also consume fruit less frequently. However, in high focus states, they consume vegetables more frequently than NBP women, pointing to a slight nutritional advantage.

In relation to our hypotheses, we found no evidence that breastfeeding women were advantaged in their access to food in the household, and, by way of comparison, only mixed evidence of an advantage for pregnant women. Particularly concerning are fruit and vegetable consumption: these food items are important sources of vital micronutrients, such as iron, folic acid, and dietary fibre, but breastfeeding women were not significantly more likely to receive fruit and vegetables than their NBP counterparts, and pregnant women were actually disadvantaged in their access to fruit. This disadvantage may potentially point to concerns about gestational diabetes given the high sugar content of fruit. However, fruit is an important source of antioxidants and micronutrients such as vitamins A and C, and reduced consumption of fruit during pregnancy may have a detrimental impact on both maternal and foetal health via micronutrient deficiencies. Further research is needed to understand why pregnant women consume less fruit. Similarly, pregnant and breastfeeding women were disadvantaged in their access to milk, which provides an important source of calcium. Additional research is needed to uncover whether similar patterns hold for other dairy products.

Our data indicate that while breastfeeding women do not consume food items more frequently than NBP women (and are in fact disadvantaged in some cases), pregnant women do experience some small advantages. This is consistent with two (potentially concurrent) possibilities. One is that households are aware of, and so prioritise, the needs of breastfeeding women, but choose to hierarchically prioritise the nutritional needs of women in the household, with the needs of pregnant women placed over breastfeeding mothers. The second is that, due to their greater nutritional needs compared to NBP women, pregnant women are responding to greater hunger, and are proactively seeking out additional nutrition. These possibilities are not mutually exclusive, and women who are both vocal about their nutritional needs and whose needs are recognized within the household context are likely to gain the greatest advantage.

In our full, nationally representative sample of breastfeeding women, we found that sociodemographic characteristics, particularly caste, religion, and vegetarianism, are important predictors of nutrition. These findings may have relevance in other settings as well. There is a strong association between social and economic deprivation and food insecurity across a wide variety of settings. While caste is a system of stratification unique to India, it is strongly correlated with socioeconomic inequalities. Our findings show that women in deprived castes were more likely than those with no caste or tribe to consume food items covered by food subsidy programmes in India, such as pulses, compared to those with no caste or tribe. However, it is not possible with these data to test here whether food security programs may mitigate or exacerbate inequalities within and between castes. Food security is an issue not only in low- and middle-income countries, but in high-income countries as well [34], [35], [36]; our results suggest that an examination of the nutritional vulnerability of low-income and ethnic minority women in all of these settings is merited.

We found no evidence in our matched-pair subsample that breastfeeding women's nutritional needs are being prioritized within the household. There has been an expansion of maternal and child health programmes in India since 2005, but empirical evidence on the impact of these programmes has been sparse, particularly in reference to safeguarding nutritional adequacy amongst vulnerable women. There have been some uneven improvements in maternal and child health across this period, but not to the extent expected in light of economic growth and expansion of these programmes. In the meantime, there has been a sharp rise in food prices corresponding to the global financial crisis, which has been associated with a reversal in progress on child malnutrition markers [37].

The evidence of active disadvantage in milk we found for both breastfeeding and pregnant women occurred in low- but not high-focus NRHM states, which may point to the efficacy of programs targeting MCH in improving household food security and knowledge about the nutritional value of dairy for women. However, given that breastfeeding women were not advantaged in any food items across multiple comparisons, we suggest that these programmes should also place additional emphasis on breastfeeding women's nutritional needs. Our study provides some evidence that the importance of fruit, vegetable, and protein consumption should be stressed in public health campaigns and food aid programmes. This may be particularly important for breastfeeding vegetarians, a sizeable minority in India, who were substantially disadvantaged in their consumption of milk and eggs, raising concerns about adequacy of protein consumption.

While we have examined the role of the NRHM, several country-level programmes in India more directly target income and food security, including the Midday Meal Scheme, which provides meals to school children; the National Rural Employment Guarantee, which guarantees work or provides income replacement in rural areas; the Public Distribution System, which provides subsidies for staple food items; and the Integrated Child Development Services Scheme, which offers a variety of healthcare and nutrition services for children and pregnant and lactating women, including nutritional supplementation. Generally, food security programmes are available in all states, most frequently on a means-tested basis. However, empirical evidence on the effectiveness of these programmes has been sparse, and the limited evidence available suggests the programmes have not been effective at improving nutrition [38]. Moreover, programmatic evidence tends to focus on children, with less attention paid to maternal nutrition and health, particularly outside of pregnancy. In the most economically deprived states in India, labelled Empowered Action Group states, poverty rates tend to be much higher, and such programmes may not be effective as they are not adequately funded to meet the needs of all vulnerable groups [38]. However, evidence on food security programmes from other countries, such as the Women, Infants, and Children programme in the US, suggest that supplemental nutrition programmes can be effective in improving nutritional intake among mothers [39]. Our findings suggest that expansion of programmes targeting food and nutrition security for breastfeeding women is needed. Future research is needed to understand the role of these programs in ensuring maternal health.

### Limitations

Although we employed stringent matched-pair multilevel models to examine women's food consumption within households, as with all observational studies, there are several limitations. First, the NFHS, the only source of representative national data of this nature on women of reproductive age, implements a food frequency questionnaire. This enables us to measure the likelihood of consumption of a variety of food items, but not the quantity or quality of the food items. It is possible that breastfeeding women consume greater quantities of meat, for example, but with similar frequency as NBP women. While it is not possible with the NFHS data, it would be of great interest to examine quantity of consumption, and for a wider range of food items; future surveys should include more detailed nutritional information. A direct measure of vegetarianism should also be included in future surveys. We were able to construct a measure of vegetarianism, which was significantly related to frequency of food consumption for breastfeeding women, based on food item consumption. However, there are many kinds of vegetarianism in India, and it is possible that some vegetarians who did not consume certain food items did so not because of a dietary disadvantage, but rather due to an active choice. For example, data from the National Sample Survey Office Consumer Expenditure Reports suggests that many vegetarians do not consume eggs or meat, but may still consume large quantities of dairy [40], [41]. While this phenomenon is likely to be randomly distributed among breastfeeding, pregnant, and NBP women, a closer examination of nutritional intake of vegetarian breastfeeding women is an important area for future research.

Second, to our knowledge, there is no specific, empirically-tested international guidance on the appropriate levels of additional caloric intake for breastfeeding women. This limited our ability to benchmark what is adequate. Future research is needed to identify the minimum needed dietary intake, including both calories and macro- and micro-nutrients, to deliver the putative benefits of breastfeeding to both mothers and children. Third, as the study relies on self-report data, it is possible that recall bias would result in consumption being over- (or under-) stated; previous medical studies, for example, have documented systematic recall bias between prenatal and postnatal women in reporting on health problems [42]. While it is unclear to what extent similar systematic bias can be expected for food frequency questionnaires, results should be interpreted with caution.

Fourth, the NRHM launched in April of 2005 [30], and the first NFHS interviews occurred in December of that year. Assuming immediate roll-out of the programme, most interviews occurred within ~1 year of the programme's launch. Any lagged effects of the programme would not be picked up here. For instance, knowledge of and cultural norms around breastfeeding practices may change slowly as a result of the Village Health and Nutrition Days, resulting in progressive improvements for women in high-focus states, which may not be picked up only 1 year after programme implementation. The effects observed here may be greater in more recent data. While previous work suggests there have been improvements in maternal and child health (child immunization, infant mortality, healthy breastfeeding initiation practices) since the programme's implementation, results around exclusive breastfeeding and care-seeking have been mixed [43], [44] – while some studies have found a positive effect, others have documented a neutral or even negative effect of the programme. Moreover, to our knowledge, no previous studies have examined women's nutrition during pregnancy and breastfeeding in association with the program. More recent data on this subject are needed.

Fifth, while we examined a wide range of potential sociodemographic correlates of breastfeeding women's nutrition, there are likely to be factors that shape nutrition that we were unable to model here. For example, exclusivity of breastfeeding, which is not directly measured in the NFHS, and BMI, which is not collected for women who have recently given birth, may be important correlates of maternal nutrition. Similarly, state level factors, such as the price of fish in coastal versus inland areas, may also impact women's consumption; while we addressed this in the full sample models, due to the matched design of the comparative study, we were unable to model individual state effects across the 29 states and territories included in the NFHS-3. Additional investigation of these factors is needed. Finally, although we examine differences by NRHM focus, control for State Domestic Product and household sociodemographics, and apply a robust research design, we are not able to directly assess how limited household resources impact access to nutrition in the household, particularly in the broader context of rising food prices in India.

### Conclusions

In sum, adequate dietary intake during lactation is vital for ensuring both maternal and child health, yet campaigns promoting increased breastfeeding have failed to examine whether women are receiving a sufficient nutritional advantage to support their greater caloric and nutrient needs as compared to NBP women. Much of the extant research on maternal nutrition during breastfeeding focuses on implications for infant health, with inadequate attention paid to maternal health. While this is an important aspect of breastfeeding, additional attention is due directly to women's health during this time. Previous evidence has suggested that malnutrition increases with multiple periods of pregnancy and lactation, representing a source of cumulative disadvantage [45], [46]. We contend that recent efforts to advance breastfeeding through public education, advocacy, and social mobilization campaigns spearheaded by national and international agencies may also need to place priority on women's nutrition during breastfeeding.

## Figures and Tables

**Table 1:** Descriptive statistics for breastfeeding, NBP, and pregnant women, matched sample, NFHS-3.

	Breastfeeding			Pregnant			NBP		
N	Mean/%	St. Dev	N	Mean/%	St. Dev	N	Mean/%	St. Dev
Age in years	1314	23.69	4.72	463	21.74	4.32	1625	22.60	5.33
Parity	1314	1.82	1.12	463	0.60	0.92	1625	0.59	1.11
Married	1314	0.98	0.14	463	0.99	0.09	1625	0.40	0.49
Education in years	1314	6.80	5.02	463	6.95	5.14	1625	7.22	4.98
Household wealth index	1314	3.60	1.29	463	3.56	1.32	1625	3.61	1.30
Urban residence	1314	0.38	0.49	463	0.42	0.49	1625	0.40	0.49
Religion									
Hindu	951	0.72	0.45	315	0.68	0.47	1149	0.71	0.46
Muslim	232	0.18	0.38	91	0.20	0.40	296.9	0.18	0.39
Christian	70	0.05	0.22	27	0.06	0.24	89	0.05	0.23
Other religion	61	0.05	0.21	30	0.07	0.25	89	0.05	0.23
Caste									
Other/No caste	469	0.36	0.48	155	0.33	0.47	580.4	0.36	0.48
Scheduled caste	218	0.17	0.37	97	0.21	0.41	277.5	0.17	0.38
Scheduled tribe	148	0.11	0.32	58	0.13	0.33	194.5	0.12	0.32
Other backward caste	479	0.36	0.48	153	0.33	0.47	572.6	0.35	0.48
Vegetarian	1314	0.31	0.46	463	0.29	0.45	1625	0.31	0.46
Frequency of consumption									
Milk	1314	1.64	1.02	463	1.66	0.99	1625	1.71	1.05
Pulses	1314	1.52	0.66	463	1.52	0.63	1625	1.55	0.67
Vegetables	1314	1.42	0.61	463	1.39	0.61	1625	1.40	0.62
Fruit	1314	2.28	0.84	463	2.11	0.88	1625	2.30	0.81
Eggs	1314	1.65	1.27	463	1.68	1.23	1625	1.69	1.27
Fish	907	1.49	1.28	329	1.62	1.28	1121	1.46	1.29
Meat	907	1.73	1.32	329	1.79	1.30	1121	1.71	1.32

## Footnotes

1 Caste is a system of social stratification unique to India. The groups known collectively as scheduled castes and scheduled tribes are officially recognized by the Indian government as being the most socially and economically disadvantaged groups [29]. Though legally banned, the caste system continues to have lasting effects. For example: children from scheduled castes and tribes face greater mortality risks than those from other castes; women in scheduled tribes have the highest total fertility rates; anaemia rates are higher among those from scheduled castes and tribes; and contraceptive use is highest among women who do not belong to any caste or tribe.
