# Supplementary material for: Breastfeeding, pregnant, and non-breastfeeding nor pregnant women's food consumption: A matched within-household analysis in India
Source: Sex Reprod Healthc. 2016 Mar;7:70–7. doi: 10.1016/j.srhc.2015.11.007 (PMC4744087; doi:10.1016/j.srhc.2015.11.007)
Supplement: Appendix S1 — Tables S1–S4. [file mmc1.docx]

**Web Appendix**

Web Table 1. High and low focus NRHM states.

| NRHM High Focus | NRHM Low Focus |
| --- | --- |
| *Bihar | Andhra Pradesh |
| *Jharkhand | Gujarat |
| *Madhya Pradesh | Delhi |
| *Rajesthan | Kerala |
| *Uttarakhand | Punjab |
| *Chhattisgarh | Karnataka |
| *Odisha | Goa |
| *Uttar Pradesh | Haryana |
| Himachal Pradesh | West Bengal |
| Jammu & Kashmir | Maharashtra |
| Arunachal Pradesh | Tamil Nadu |
| Meghalaya | Dadra & Nagar Haveli |
| Manipur | Daman & Diu |
| Tripura | Telangana |
| Assam | Puducherry |
| Mizoram | Chandigarh |
| Nagaland | Andaman & Nicobar Islands |
| Sikkim | Lakshadweep |

Notes: * Denotes EAG state; Dadra & Nagar Haveli, Daman & Diu, Telangana, Puducherry, Chandigarh, Andaman & Nicobar Islands, and Lakshadweep were not sampled in the NFHS

Web Table 2. Multilevel linear models of sociodemographic characteristics and nutritional intake for breastfeeding women, all states, NFHS-3

|  | *Milk* | *Pulses* | *Veg* | *Fruit* | *Eggs* | *Fish* | *Meat* |
| --- | --- | --- | --- | --- | --- | --- | --- |
| Child’s Age (Months) | 0.00^***^ (0.00) | -0.00 (0.00) | -0.00 (0.00) | 0.00 (0.00) | 0.00 (0.00) | 0.00 (0.00) | 0.00 (0.00) |
| Age (Years) | -0.00 (0.00) | 0.00 (0.00) | -0.00 (0.00) | -0.00^*^ (0.00) | -0.00 (0.00) | -0.00^*^ (0.00) | -0.00 (0.00) |
| Vegetarian | -0.22^***^ (0.02) | 0.01 (0.01) | 0.01 (0.01) | -0.08^***^ (0.02) | -2.09^***^ (0.02) | 0.00 (.) | 0.00 (.) |
| Parity | 0.01 (0.01) | 0.01^***^ (0.00) | 0.01^**^ (0.00) | 0.01 (0.00) | 0.01 (0.00) | 0.02^***^ (0.01) | 0.02^***^ (0.00) |
| Married | -0.06 (0.06) | -0.06 (0.04) | -0.02 (0.04) | -0.00 (0.05) | -0.05 (0.05) | -0.03 (0.05) | -0.06 (0.04) |
| Education (Years) | -0.01^***^ (0.00) | -0.01^***^ (0.00) | -0.01^***^ (0.00) | -0.02^***^ (0.00) | -0.00 (0.00) | -0.01^**^ (0.00) | -0.01^***^ (0.00) |
| Urban | 0.04^*^ (0.02) | -0.04^**^ (0.01) | -0.00 (0.01) | -0.09^***^ (0.01) | 0.00 (0.02) | -0.04^*^ (0.02) | -0.06^***^ (0.01) |
| Wealth Index | -0.05^***^ (0.01) | -0.07^***^ (0.00) | -0.05^***^ (0.00) | -0.05^***^ (0.01) | -0.02^***^ (0.01) | -0.05^***^ (0.01) | -0.05^***^ (0.01) |
| Muslim | 0.10^***^ (0.03) | 0.10^***^ (0.01) | 0.01 (0.01) | -0.01 (0.02) | 0.01 (0.02) | -0.05^*^ (0.02) | -0.11^***^ (0.02) |
| Christian | -0.17^***^ (0.04) | 0.07^**^ (0.03) | -0.10^***^ (0.02) | 0.02 (0.03) | 0.03 (0.03) | 0.17^***^ (0.03) | 0.02 (0.03) |
| Other religion | -0.03 (0.04) | 0.11^***^ (0.03) | -0.03 (0.02) | -0.02 (0.03) | -0.01 (0.03) | -0.08^*^ (0.04) | -0.05 (0.03) |
| Scheduled Caste | 0.11^***^ (0.02) | 0.04^**^ (0.01) | 0.01 (0.01) | 0.03 (0.02) | 0.07^***^ (0.02) | -0.02 (0.02) | 0.01 (0.02) |
| Scheduled Tribe | -0.18^***^ (0.03) | 0.11^***^ (0.02) | 0.03^*^ (0.02) | -0.05^*^ (0.02) | 0.07^**^ (0.02) | 0.08^**^ (0.02) | -0.02 (0.02) |
| Other Backward Caste | 0.00 (0.02) | 0.04^**^ (0.01) | -0.01 (0.01) | 0.05^**^ (0.02) | 0.01 (0.02) | 0.02 (0.02) | -0.00 (0.02) |
| Constant | 1.79^***^ (0.09) | 2.44^***^ (0.05) | 1.60^***^ (0.05) | 2.56^***^ (0.07) | 2.14^***^ (0.07) | 2.41^***^ (0.08) | 2.63^***^ (0.06) |

Notes: Standard errors in parentheses; ^*^ *p* < 0.05, ^**^ *p* < 0.01, ^***^ *p* < 0.001; models include dummies for 28 states and union territories.

Web Table 3. Multilevel linear regression models of frequency of fruit consumption, all states, matched sample, NFHS-3

|  | B (SE) |
| --- | --- |
| Currently Breastfeeding | -0.04 (0.04) |
| Vegetarian | 0.02 (0.04) |
| Age (Years) | -0.00 (0.00) |
| Parity | 0.01 (0.02) |
| Married | -0.02 (0.05) |
| Education (Years) | -0.02^***^ (0.00) |
| Urban | -0.16^***^ (0.04) |
| Wealth Index | -0.09^***^ (0.02) |
| Religion (Hindu ref) |  |
| Muslim | -0.07 (0.06) |
| Christian | 0.00 (0.09) |
| Other Religion | -0.06 (0.08) |
| Caste (No/other ref) |  |
| Scheduled Caste | 0.09 (0.05) |
| Scheduled Tribe | 0.07 (0.07) |
| Other Backward Caste | 0.15^***^ (0.04) |
|  |  |
| Constant | 2.88^***^ (0.11) |

Notes: *p<.05 **p<.01 ***p<.001; NBP is women who are neither pregnant nor breastfeeding

Web Table 4. Multilevel linear regression models of food consumption by NRHM focus and breastfeeding or pregnancy status, including State Domestic Product as a control, matched sample, NFHS-3

|  | **Model 1** | |  | **Model 2** | |  | **Model 3** | |  | **Model 4** | |
| --- | --- | --- | --- | --- | --- | --- | --- | --- | --- | --- | --- |
|  | *Breastfeeding vs NBP, Low Focus* | |  | *Breastfeeding vs NBP, High Focus* | |  | *Pregnant vs. NBP, Low Focus* | |  | *Pregnant vs. NBP, High Focus* | |
| Milk | -0.14^*^ | (0.07) |  | 0.08 | (0.06) |  | -0.33^**^ | (0.12) |  | 0.09 | (0.12) |
| Pulses | -0.01 | (0.04) |  | 0.01 | (0.04) |  | 0.04 | (0.07) |  | -0.07 | (0.07) |
| Vegetables | -0.03 | (0.04) |  | 0.02 | (0.03) |  | -0.06 | (0.08) |  | 0.13^*^ | (0.06) |
| Fruit | -0.07 | (0.06) |  | -0.03 | (0.05) |  | -0.04 | (0.11) |  | -0.14 | (0.08) |
| Eggs | -0.11 | (0.06) |  | 0.02 | (0.06) |  | -0.07 | (0.13) |  | 0.18 | (0.1) |
| Fish | 0.02 | (0.08) |  | -0.06 | (0.07) |  | 0.28 | (0.15) |  | 0.09 | (0.11) |
| Meat | 0.07 | (0.07) |  | -0.04 | (0.06) |  | -0.12 | (0.12) |  | 0.06 | (0.09) |

Notes: *p<.05 **p<.01 ***p<.001; standard errors in parentheses; NBP is women who are neither pregnant nor breastfeeding
